# Supplementary material for: Human-Biting Ixodes scapularis Submissions to a Crowd-Funded Tick Testing Program Correlate with the Incidence of Rare Tick-Borne Disease: A Seven-Year Retrospective Study of Anaplasmosis and Babesiosis in Massachusetts
Source: Microorganisms. 2023 May 27;11(6):1418. doi: 10.3390/microorganisms11061418 (PMC10301449; doi:10.3390/microorganisms11061418)
Supplement: Supplementary file 1 [file microorganisms-11-01418-s001.zip › microorganisms-2408317-supplementary.pdf]

Table S1: Molecular methods used for the identification of *Ixodes scapularis* ticks and the detection of *Babesia microti* and *Anaplasma phagocytophilum*.

Target gene	*Application*	*Type*	*Sequences (5′-3′)*	*Tm (C)*
ITS	*Ixodes scapularis* identification	Forward	TGCGTTTTCTTTGAGCAAATGCACGAG	60
		Reverse	GTACGGGATTTTCCACAAACGGTATCCA	
		Probe	TGCGCTTAACCAGTCCTCCTCCTCCTACGA	
Tubulin	*Babesia* detection	Forward	GATTTGGAACCTGGCACCATG	60
		Reverse	AATGACCCTTAGCCCAATTATTTCC	
		Probe	ATCTGGCCCATACGGTGAATTGTTTCGC	
MSP-2	*Anaplasma* detection	Forward	ATGGAAGGTAGTGTTGGTTATGGTATT	60
		Reverse	TTGGTCTTGAAGCGCTCGTA	
		Probe	TGGTGCCAGGGTTGAGCTTGAGATTG	

Table S2. Characteristics of tick attachment.

	Larvae	Nymphs	Adults
Mean attachment time to victim (hours)	17.33	17.28	20.98
Engorgement rate, percentage of ticks (laboratory determined)	9.77	10.80	10.00
Mean age of victim (years)	26	34	41
Reported attachment location (percentage of ticks):			
Arm, armpit, back, chest, ear, head, neck, shoulder, stomach	44.9	49.1	62.5
Buttocks, groin, foot, leg, knee	48.2	41.1	28.3
Other, not provided	6.9	9.8	9.2

Table S3: Spearman's rho with 95% confidence intervals for correlations between tick measures and human disease.

	2015	2016	2017	2018	2019	2020	2021	aggregated
Human anaplasmosis								
All sub	0.655 [0.544,0.729]	0.668 [0.575,0.744]	0.622 [0.537,0.695]	0.678 [0.620,0.741]	0.625 [0.540,0.697]	0.695 [0.622,0.755]	0.720 [0.651,0.778]	0.830 [0.800,0.856]
Inf sub	0.541 [0.309,0.711]	0.672 [0.476,0.805]	0.590 [0.419,0.721]	0.683 [0.554,0.780]	0.722 [0.597,0.812]	0.707 [0.599,0.790]	0.650 [0.477,0.775]	0.732 [0.665,0.789]
adult	0.700 [0.603,0.777]	0.727 [0.640,0.795]	0.625 [0.536,0.700]	0.688 [0.613,0.751]	0.670 [0.590,0.737]	0.701 [0.629,0.761]	0.728 [0.656,0.789]	0.713 [0.654,0.764]
Nymph	0.791 [0.732,0.838]	0.622 [0.465,0.741]	0.617 [0.490,0.718]	0.660 [0.555,0.744]	0.735, [0.647,0.803]	0.684 [0.582,0.764]	0.759 [0.678,0.821]	0.708 [0.643,0.762]
Human babesiosis								
All sub	0.584 [0.454,0.690]	0.667 [0.548,0.759]	0.594 [0.480,0.689]	0.634 [0.531,0.728]	0.633 [0.531,0.717]	0.614 [0.500,0.706]	0.712 [0.631,0.786]	0.597 [0.515,0.667]
Inf sub	0.773 [0.656,0.889]	0.407 [0.06,0.664]	0.642 [0.456,0.774]	0.722 [0.563,0.823]	0.517 [0.322,0.670]	0.609 [0.434,0.740]	0.710 [0.505,0.834]	0.584 [0.485,0.669]
adult	0.591 [0.455,0.700]	0.596 [0.461,0.704]	0.586 [0.473,0.680]	0.581 [0.469,0.675]	0.567 [0.457,0.660]	0.580 [0.471,0.672]	0.684 [0.592,0.758]	0.552 [0.465,0.629]
Nymph	0.661 [0.502,0.776]	0.644 [0.460,0.775]	0.667 [0.536,0.767]	0.753 [0.680,0.810]	0.675 [0.560,0.765]	0.722 [0.613,0.803]	0.753 [0.650,0.838]	0.684 [0.611,0.745]
